# Vestibular and oculomotor influences on visual dependency

**DOI:** 10.1152/jn.00895.2015

**Published:** 2016-06-29

**Authors:** R. Edward Roberts, Mariane Da Silva Melo, Aazim A. Siddiqui, Qadeer Arshad, Mitesh Patel

**Affiliations:** ^1^Neuro-otology Unit, Division of Brain Sciences, Imperial College London, London, United Kingdom;; ^2^Federal University of Minas Gerais, Belo Horizonte, Minas Gerais, Brazil; and; ^3^School of Biosciences, University of East London, London, United Kingdom

**Keywords:** visual dependency, field dependence-independence, subjective visual vertical, vestibular activation, ocular torsion

## Abstract

The degree to which a person relies on visual stimuli for spatial orientation is termed visual dependency (VD). VD is considered a perceptual trait or cognitive style influenced by psychological factors and mediated by central reweighting of the sensory inputs involved in spatial orientation. VD is often measured with the rod-and-disk test, in which participants align a central rod to the subjective visual vertical (SVV) in the presence of a background that is either stationary or rotating around the line of sight—dynamic SVV. Although this task has been employed to assess VD in health and vestibular disease, what effect torsional nystagmic eye movements may have on individual performance is unknown. Using caloric ear irrigation, 3D video-oculography, and the rod-and-disk test, we show that caloric torsional nystagmus modulates measures of VD and demonstrate that increases in tilt after irrigation are positively correlated with changes in ocular torsional eye movements. When the direction of the slow phase of the torsional eye movement induced by the caloric is congruent with that induced by the rotating visual stimulus, there is a significant increase in tilt. When these two torsional components are in opposition, there is a decrease. These findings show that measures of VD can be influenced by oculomotor responses induced by caloric stimulation. The findings are of significance for clinical studies, as they indicate that VD, which often increases in vestibular disorders, is modulated not only by changes in cognitive style but also by eye movements, in particular nystagmus.

## Abstract (precis)

*Participants made verticality judgments using the rod-and-disk test, a test of visual dependence, and then repeated after caloric irrigation. If the combination of rotating disk and caloric increased the slow-phase velocity of the torsional nystagmus the tilt in subjective verticality increased, whereas reductions in eye velocity were associated with reduced tilt. Thus visual dependency measures are not only modulated by perceptual style but can also reflect local vestibulo-ocular function, specifically torsional eye movements*.

## NEW & NOTEWORTHY

*Participants made verticality judgments using the rod-and-disk test, a test of visual dependence, and then repeated after caloric irrigation. If the combination of rotating disk and caloric increased the slow-phase velocity of the torsional nystagmus the tilt in subjective verticality increased, whereas reductions in eye velocity were associated with reduced tilt. Thus visual dependency measures are not only modulated by perceptual style but can also reflect local vestibulo-ocular function, specifically torsional eye movements*.

in healthy individuals spatial orientation depends upon the integration of vestibular, visual, and somatosensory cues (Brandt and Dieterich 1999; Bronstein 1999; Lopez et al. 2005b). However, the relative weighting afforded to each of these systems is known to vary across the normal population. A specific example of this heterogeneity is the degree to which a person relies on his/her vision to make judgments about gravitational vertical. Witkin and Asch first reported that when participants were presented with visual cues to verticality that were tilted with respect to true gravitational vertical (e.g., an entire room with furniture set at an angle of 22°), a proportion of the population incorrectly estimated that they themselves were also tilted with respect to true vertical in the direction of the visual reference frame tilt (Witkin and Asch 1948). This effect has been replicated in subsequent studies employing a simpler “rod-and-frame” arrangement in which the rod is adjusted by the participant to what he/she perceives as vertical (subjective visual vertical, SVV) in the presence of a static tilted frame (Guerraz et al. 2001; Lopez et al. 2006; Witkin and Asch 1948).

The influence that moving visual stimuli have on the perception of verticality was explored in a seminal paper aptly titled “Moving visual scenes influence the apparent direction of gravity” (Dichgans et al. 1972). This established one of the most frequently employed designs used to measure the effect of visual motion on gravitational verticality judgments, an optokinetic disk rotating in the roll (frontal) plane as the visual stimulus—the rod-and-disk test (Bronstein et al. 1996; Dichgans et al. 1972; Guerraz et al. 2001). Here participants initially align a rod to what they perceive to be gravitational vertical against a stationary background with no cues to verticality—a measure of the SVV. They then repeat the task against a background of optokinetic disk roll motion; this has been termed dynamic SVV (Dieterich and Brandt 1993). The effect of the roll motion is to bias estimates of verticality in the direction of motion. The difference in tilt between the static SVV and dynamic SVV conditions is referred to here as visual dependency (VD), i.e., the change in tilt associated with a moving visual background compared with a static baseline.

The impact of vestibular activation on perceived verticality has been explored with rotational and brain stimulation in healthy individuals (Mars et al. 2001; Pavlou et al. 2003) and in patients with peripheral or central disorders (Bronstein et al. 2003; Friedmann 1970; Lopez et al. 2007). Most frequently this has focused on changes in SVV in patients with unilateral loss (Bisdorff et al. 1996; Böhmer and Mast 1999; Brandt et al. 1994; Curthoys et al. 1991; Halmagyi et al. 1979; Riordan-Eva et al. 1997). In contrast, there have been few investigations into the effect that vestibular activation has upon measurements of dynamic SVV and VD. This is in part because of a tendency to treat this effect as a purely psychological trait or cognitive style (Evans et al. 2013). Accordingly, until recently comparatively little research has been focused on its biological basis (Bronstein et al. 1996; Hao et al. 2013; Walter and Dassonville 2011). This is somewhat surprising given that VD is often increased in patients with chronic vestibular symptoms (Agarwal et al. 2012; Guerraz et al. 2001) and recent reports have suggested that VD could be a strong predictor of clinical outcome following acute vestibular lesions such as vestibular neuritis (Cousins et al. 2014). However, as such patients exhibit a resting nystagmus in the acute phase with both horizontal and torsional components (Strupp and Brandt 2009), it is unclear whether such eye movements can also affect the measurement of VD. Two studies have provided evidence to suggest a connection between VD and torsional eye movements. In separate reports, Lopez et al. examined either dynamic SVV or torsional optokinetic nystagmus in two groups of patients before and after unilateral vestibular neurectomy (Lopez et al. 2005a, 2007). In the first report the authors observed significant asymmetry in the torsional eye movements in response to optokinetic stimulation rotating around the line of sight, with a bias toward the ipsilesional side. In the second study they found substantially increased tilt in the dynamic SVV task during the acute stage following unilateral vestibular neurectomy, suggesting that the increased torsion and bias in SVV might be connected.

In this study we aimed to build on the work of Lopez et al. primarily by exploring how changes in ocular torsion can bias measures of VD in healthy participants. Lopez et al. studied participants after vestibular neurectomy, which induces a permanent deficit in patients with variable degrees of preexistent vestibular dysfunction. In contrast, here we used caloric-induced vestibular nystagmus, which can be repeated in the same subject with normal baseline vestibular function. Apart from the scientific interest in ascertaining how eye movements influence verticality estimates, the results of these studies should contribute to the understanding of VD in a common vestibular disorder called vestibular neuritis. Given the orientation of the semicircular canals and additional thermal effects on the labyrinth, the caloric stimulus induces nystagmus not only through its main activation of the horizontal canal (horizontal component) but also by stimulating the vertical canals and otoliths (torsional components) (Yagi et al. 1992), thus simulating the eye movements and vestibular imbalance present during acute vestibular neuritis. In the present study we examined how the direction of visual motion rotation influenced measures of VD with the rod-and-disk test and then recorded eye movements in a group of normal subjects in order to specifically explore the contribution of ocular torsion to these judgments. Our hypothesis was that the caloric-induced torsional eye movements would interact with the direction of the roll motion stimulus to either increase or decrease subjective verticality, contingent on the relative direction of slow-phase velocity eye movements induced by each stimulus.

## METHODS

### Subjects

Fifty-two healthy volunteers (mean age = 22.5 yr, SD = 2.4 yr) participated in this study in total: 8 each in *experiments 1–4*, 12 in *experiment 5*, and 20 in *experiment 6* (includes participants from *experiment 5*). Participants had no history of labyrinthine or neurological disorders. All subjects were naive to the purpose of the study and provided written informed consent to participate after reviewing the protocol. The study was approved by the local research ethics committee.

### Apparatus

**Fig. 1.**
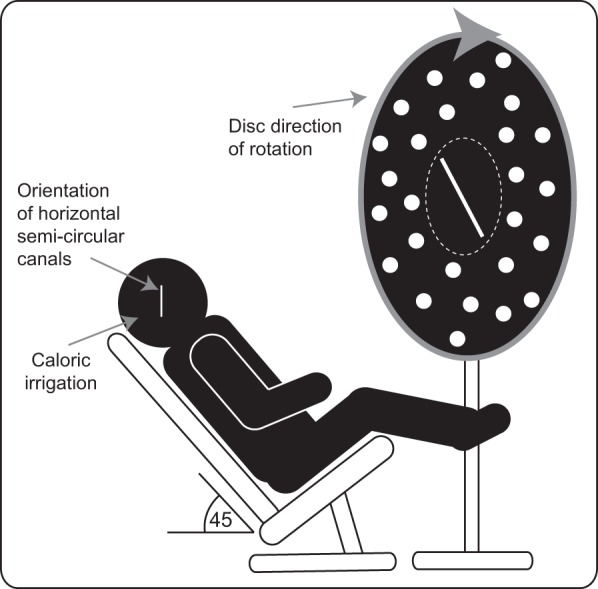
Experimental apparatus. Participants were seated at an angle of 45° to ensure maximal stimulation of the horizontal semicircular canals during caloric irrigation. The small central disk containing the straight line overlapped the dots on the large disk, so that the line was never traversed by the moving dots.

The participants lay supine on a couch tilted up by 45° to evoke maximal stimulation of the horizontal semicircular canals, with the head tilted ∼30° off vertical (Coats and Smith 1967). A head rest and strap were used to maintain head position (Fig. 1). A vertical frontal (roll) plane optokinetic rotating disk (diameter of 0.9 m), which was black and covered with randomly placed luminous dots, was positioned 80 cm directly in front of the participants. When viewed at this distance it subtended an angle of ±30°. The height of the disk was adjusted such that the center was in line with primary gaze. The disk could be rotated either clockwise or anticlockwise at 30°/s around the subjects' line of sight in the roll plane. At its center was a black disk with a 0.1-m luminous rod (7° visual angle) that subjects had to fixate so that they could adjust the tilt of the line, via a potentiometer, to what they perceived to be vertical. This secondary black disk covered the underlying main disk so that the line was never traversed by the moving dots; this arrangement also prevented small (<7°) deviations of gaze away from the straight line during the task leading to the eyes being driven away by the optokinetic stimulus. Analysis of participants' average gaze position indicated that they were able to maintain fixation within the region of the central disk during 95% of task. Subjects performed eight trials for each condition. At the beginning of the experiment an initial eye movement calibration was performed. At the beginning of each trial the rod was set to ±40° from vertical. The rod tilt for each trial was recorded as the difference in degrees between true vertical and the subjects' final placement of the rod. Eye position of both eyes was recorded (250 Hz) with the head-mounted video oculography system C-ETD 3706 (Chronos Vision, Berlin, Germany) in a head-fixed frame of reference. Peak velocity and maximum peak-to-peak amplitude of the slow-phase eye movements were calculated by fitting a linear slope to the eye position trace with in-house Analysis software (David Buckwell). Data were analyzed with SPSS version 22, using paired *t*-tests or ANOVA where appropriate.

### Task

All participants were required to set a central line to vertical in the presence of either a stationary or a rotating background (the rod-and-disk test) during binocular viewing. VD was calculated as the mean increase in visual motion-induced tilt compared with a static background. Depending on which experiment they were taking part in, the task might be conducted during caloric-induced nystagmus. In these conditions the external auditory meatus was irrigated with water at 30°C (cold water) or 44°C (warm water) at a rate of 500 ml/min for 40 s (CHART VNG; ICS medical).

### Experiments

The aim of the following experiments was first to assess the effect that different combinations of visual motion direction (clockwise or anticlockwise) and caloric (warm or cold)-induced nystagmus would have on the perception of verticality. We then tested how these conditions influenced torsional eye movements and explored the relationship with our measure of VD.

#### Experiments 1 and 2.

We assessed how the direction of visual motion influenced perceived verticality (dynamic SVV/VD) after cold caloric stimulation. In *experiment 1*, baseline VD was first assessed with a clockwise visual stimulus and then measured again immediately after a right cold irrigation. In *experiment 2*, we used the same procedure but reversed the direction of the visual stimulus (now anticlockwise visual motion) and then remeasured after a right cold irrigation. The order of conditions was balanced across participants.

#### Experiment 3.

The direction of motion was kept the same, but this time the temperature of stimulation (and hence direction of nystagmus) was varied with either a cold or a warm caloric irrigation of the right ear.

#### Experiment 4.

As a control for the effect of caloric stimulation on SVV, *experiment 4* tested for deviations in SVV before and after a cold caloric irrigation of the right ear in the presence of a stationary background with no cues to verticality (viewing the stationary disk).

In the final experiments torsional eye movements were recorded in a group of normal subjects to explore the role of ocular torsion in this process. Our hypothesis was that vestibular and optokinetic-induced torsional eye movements would interact to increase or decrease in degree of tilt in verticality judgments, dependent on the relative direction of slow-phase velocities.

#### Experiment 5.

VD was measured without caloric stimulation and after right cold irrigation and right warm irrigation.

#### Experiment 6.

VD was measured during visual motion alone, caloric alone, and visual motion + right cold caloric, and changes in tilt between the conditions were related to changes in ocular torsion and amplitude.

## RESULTS

**Fig. 2.**
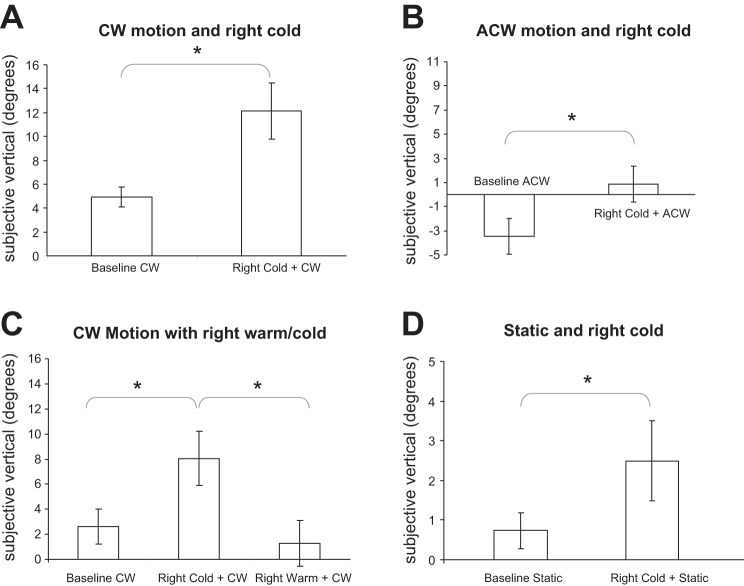
Changes in visual dependency and SVV (±SD) in *experiments 1–4*. *A*: cold caloric irrigation of the right ear significantly increased (*) tilt during clockwise (CW) visual motion. *B*: cold caloric of the right ear was associated with a significant reduction (*) in VD during anticlockwise (ACW) motion. *C*: cold caloric of the right ear significantly increased (*) the degree of tilt, but a warm caloric showed a trend toward reduction in tilt that was not significantly different from baseline. *D*: right cold caloric significantly increased (*) SVV estimates in the presence of a static visual field. Visual dependency was computed as SVV during disk roll motion − SVV during disk stationary.

In *experiment 1*, the slow phases of the torsional nystagmus from the visual stimulus and caloric stimulus were in the same direction. During clockwise visual motion, subjects tilted the line clockwise with respect to gravitational vertical as expected. There was a significant increase in the deviation from baseline (visual motion alone; mean = 4.9°, SD = 2.7°) after a cold caloric irrigation of the right ear (mean = 12.1°, SD = 7.3°) [Fig. 2*A*; paired-samples *t*-test (*t* = 3.9, *P* = 0.006)].

In *experiment 2*, the torsional nystagmus induced by the visual and caloric stimuli were in opposing directions. As expected, under anticlockwise visual motion participants tilted the line in the direction of motion away from vertical (mean = −3.5°, SD = 4.2°). After a cold caloric irrigation of the right ear, there was a significant clockwise bias back toward vertical (mean = 0.87°, SD = 4.2°) [Fig. 2*B*; paired-samples *t*-test (*t* = 5.9, *P* < 0.001)]. Thus right cold caloric irrigations biased line tilt in a clockwise direction. Hence, the direction of visual motion critically affected the subjective verticality measures (*t* = 3.8, *P* < 0.002; independent-samples *t*-test).

In *experiment 3*, we kept the direction of motion constant and changed the direction of vestibular nystagmus by using either warm or cold irrigations. A one-way repeated-measures ANOVA determined that there was a significant difference between VD for each of the three conditions [*F*(2,14) = 14.6, *P* < 0.001]. Post hoc *t*-tests revealed that compared with baseline (mean = 3.4°, SD = 3.6°) right cold irrigation (which induces eye movements congruent with the direction of disk motion) significantly increased line tilt (mean = 8.3°, SD = 4.5°, *P* = 0.017) whereas right warm irrigations (incongruent with disk motion) were associated with a reduction in tilt, but this was not statistically significant (*P* > 0.3; see Fig. 2*C*). In both cases, the line was tilted clockwise from vertical. Line deviation from true vertical following right cold irrigation was significantly greater than during right warm irrigation (*P* = 0.007). This demonstrates that during the same visual motion stimulus the direction of caloric-induced torsional nystagmus differentially affects measures of VD.

In a control experiment (*experiment 4*) we assessed separately the effect that vestibular stimulation had on SVV when the optokinetic disk was stationary. We found a significant increase in SVV in the clockwise direction compared with baseline (mean = 0.74°, SD = 0.45°) after the cold caloric irrigation (mean = 2.59°, SD = 1.01°) [paired-samples *t*-test (*t* = 2.8, *P* = 0.01); see Fig. 2*D*].

**Fig. 3.**
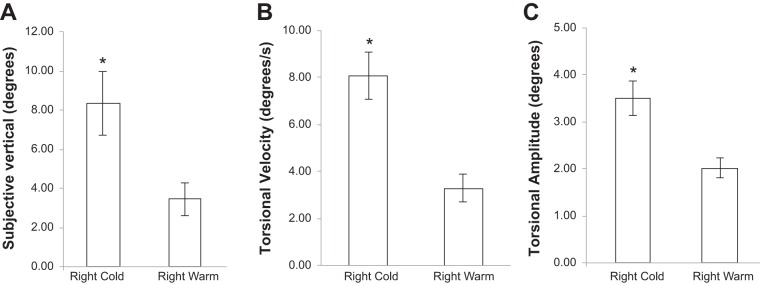
Perceived verticality, torsional velocity, and amplitudes for clockwise motion during either right cold or right warm caloric. Perceived verticality (line tilt during roll motion background; *A*), torsional velocity (*B*), and amplitudes (*C*) were all significantly higher (*) when clockwise motion was combined with a right cold caloric irrigation compared with a right warm irrigation.

In the final experiments (*experiments 5* and *6*) mean VD was 2.72° (SD = 1.42°), whereas mean VD was 4.7° (SD = 2.95°) after right warm irrigation and 6.53° (SD = 5.63°) after right cold irrigation. The mean VD during both vestibular stimuli (warm and cold) was 5.61° (SD = 2.82°). This was significantly higher than VD measured without vestibular stimulation (*t* = 3.18, *P* < 0.004; paired-samples *t*-test). We then focused on how changes in torsional eye movements were related to changes in line tilt and whether torsional velocity and amplitudes were different when clockwise visual motion was combined with either warm or cold irrigations. Torsional velocities and nystagmus amplitudes for right warm + visual motion (velocity mean = 3.31°/s, SD = 0.59°/s; amplitude mean = 2.02°, SD = 0.22°) were significantly smaller than right cold + visual motion (velocity mean = 8.09°/s, SD = 1.01°/s; amplitude mean = 3.50°, SD = 0.38°/s; *P* < 0.001), mirroring the effect described in *experiment 3* (Fig. 3, *B* and *C*).

In *experiment 6* we then investigated whether changes in the degree of line tilt were related to changes in torsional eye movements in one group of participants under three conditions (clockwise visual motion alone, right cold caloric alone, and visual motion + right cold caloric).

In terms of tilt, the caloric-alone condition was associated with the smallest degree of deviation from true vertical (mean = 2.0°, SD = 2.2°). The visual motion-alone condition induced a significantly larger tilt (mean = 4.8°, SD = 4.4°; *P* = 0.003, paired *t*-test), and the combined condition (visual motion + right cold caloric) had the largest value (mean = 9.4°, SD = 5.5°), which was significantly larger than the other conditions (*P* < 0.0001).

The torsional velocities for caloric alone (mean = 4.12°/s, SD = 2.09°/s) and visual motion alone (mean = 2.61°/s, SD = 1.9°/s) were significantly different (*P* = 0.02); however, the velocity in the combined condition was significantly higher than both (mean = 8.74°/s, SD = 3.15°/s; *P* < 0.0001). For the torsional amplitude there was no difference between visual motion alone (mean = 2.14°, SD = 1.39°) and caloric alone (mean = 2.29°, SD = 0.86°/s; *P* > 0.6), but both were significantly smaller than the combined condition (mean = 4.50°, SD = 1.63°; *P* < 0.0001). These values for optokinetic stimulation-induced torsion are consistent with previous reports (Collewijn et al. 1985; Thilo et al. 1999).

**Fig. 4.**
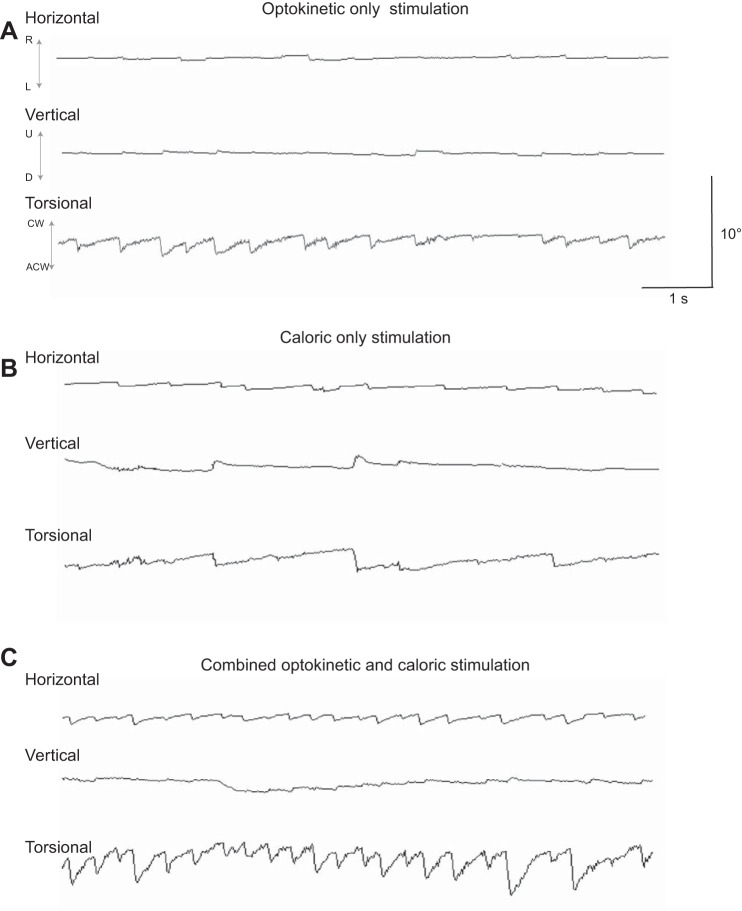
Horizontal, vertical and torsional nystagmus measurements for a representative participant. *A*: optokinetic stimulation (OKS) disk rotating clockwise. *B*: right ear cold caloric alone. *C*: combined OKS and caloric stimulation. There was significantly increased anticlockwise torsional nystagmus (north pole of eye beats toward the left shoulder) during the combined protocol compared with either visual or vestibular stimulation alone.

Interestingly, both for the degree of line tilt and torsional velocity, the algebraic sums of the visual motion-alone and caloric-alone conditions were significantly smaller than in the combined condition (perceived vertical: *P* < 0.0001; torsional velocity: *P* < 0.02). Therefore, it appears that when the stimuli synergistically interact the effect on line tilt and torsional velocity is not convincingly explained by a linear summation of the separate components. However, it is possible that the internal representation of visual tilt does not use a linear scale; therefore we summated the logs of the data for both measures. This revealed no significant difference between the contribution of separate components and the combined conditions (*P* > 0.2). This suggests that the magnitude of both torsional velocity eye movements and line tilt produced by combined visual and vestibular stimuli could reflect a nonlinear internal magnitude scale, i.e., following Fechner's scale. Traces from a representative participant are shown in Fig. 4.

**Fig. 5.**
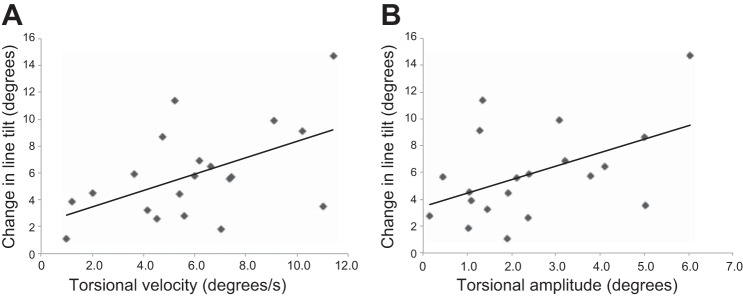
Association between change in perceived verticality and change in ocular torsion. There was a significant correlation between the increase in degree of line tilt in the combined condition (right cold caloric + clockwise disk roll motion) compared with baseline (visual dependency) for both torsional velocity (*A*) and torsional amplitude (*B*).

We then examined whether changes in torsional velocity and amplitude predicted the size of the increase in line tilt between visual motion alone and the combined condition. This revealed a significant positive correlation between the increase in degree of tilt after caloric and the increase in both torsional velocity (*r* = 0.52, *P* = 0.02; Pearson correlation) and amplitude (*r* = 0.48, *P* = 0.03) (see Fig. 5).

One possible explanation for the effects we report is that the horizontal component of nystagmus produced by the caloric stimulus may have biased these perceptual judgments. Indeed, there was a horizontal nystagmus present in the combined stimulus protocol, but the maximum vestibulo-ocular reflex slow-phase velocity was highly suppressed compared with that elicited during darkness in these participants, with values reduced to ∼25% (mean velocity = 5.7°/s, SD = 3.5°/s). We tested whether this eye movement component was predictive of the differences in baseline VD but found no significant correlation between VD and horizontal nystagmus velocity or amplitude (*P* > 0.7).

We also examined whether baseline VD during visual motion alone was predicted by the size of increase in torsional velocity observed in the caloric + visual motion condition, but we found no evidence of a significant relationship here between individual differences in visual dependence and response to vestibular challenge (*P* > 0.1). Nor was there a significant relationship between torsional velocity and tilt during optokinetic stimulus alone (*P* > 0.2), which likely reflects the known bimodal distribution of VD across the population.

## DISCUSSION

Here we investigated VD by measuring the SVV with and without a roll-plane rotating visual background (dynamic SVV). Although there is a widely held opinion that SVV tilts are selectively due to otolith asymmetry, previous work has shown this assumption to be incorrect (Pavlou et al. 2003). Pavlou et al. demonstrated that vertical semicircular canal stimulation, via yaw plane rotations with the head in different positions, was associated with changes in ocular torsion and SVV tilt. This indicates that the vertical semicircular canals, in addition to the otoliths, play a role in determining subjective gravitational-vertical. Although these findings illustrate the vestibular contribution to gravitational and SVV perception, the primary aim of the present study was to investigate the effects of torsional nystagmic eye movements on VD (in this case using the dynamic visual vertical or rod-and-disk task).

In acute vestibular failure VD also increases, but a direct contribution of nystagmus has not been confirmed (Cousins et al. 2014; Lopez et al. 2007). Acute vestibular loss has also been shown to reduce or abolish contralesional VD measured with the rod-and-frame tool (Lopez et al. 2006). In the light of our present findings, this could be interpreted as (or due to) measures of VD being biased by the direction of baseline nystagmus. In chronic stages after vestibular loss, the bias in VD is corrected by central adaptation mechanisms. Despite this, recent work has suggested that higher values of VD (rod and disk, as used here) are a strong predictor of clinical outcome following vestibular neuritis (Cousins et al. 2014), whereas the degree of vestibular loss, as measured by the 3D video head-impulse test, is not (Patel et al. 2016). Therefore, understanding the brain mechanisms underlying increased VD may have important clinical implications.

In this study we report that torsional eye movements induced by vestibular and visual stimuli interact to increase or decrease perceived verticality, as measured with the rod-and-disk test. Under conditions where the combined stimuli increased the degree of line tilt compared with baseline, we found that this change was strongly predicted by the increase in ocular torsion. We observed the largest increase in tilt when the slow phase of the caloric-induced torsional eye movement was in the same direction as that induced by the visual motion stimulus (e.g., right cold + clockwise motion). When the slow phases of the eye movements were in opposition, e.g., clockwise motion + right warm, we observed a reduction in tilt.

Although it is long established that ocular (torsional) tilt is the main factor responsible for large, abnormal tilts of the SVV (Curthoys et al. 1991; Dieterich and Brandt 1992; Pavlou et al. 2003; Wade and Curthoys 1997), it is reasonable to ask why torsional eye movements have such a strong effect on perception. One possible explanation is that, unlike horizontal and vertical eye movements, the perceptual system is largely unaware of torsional eye movements and thus perceptual constancy during torsional eye movements would be distorted (Wade and Curthoys 1997) and that torsional eye movements are generally not under volitional control (Balliet and Nakayama 1978; Brodsky et al. 1999).

Although these data point toward a significant role for ocular torsion in measurements of VD using a roll plane stimulus, it is important to acknowledge alternative interpretations. The higher overall value of torsion in the congruent condition (visual and vestibular both elicit slow-phase velocity in the same direction) could be due to the horizontal nystagmus interfering with the subjects' ability to fixate the SVV line. However, neither the peak velocity nor the amplitude of the horizontal nystagmus was correlated with baseline VD in the final experiment, the degree of horizontal nystagmus was largely visually suppressed (∼25% of peak values in the dark), and gaze position remained within the central disk area 95% of the time during task performance. In addition, we did not observe substantial modulation of SVV in the caloric-alone condition, indicating that horizontal nystagmus per se is unlikely to be a major cause of increased SVV tilt in the combined condition. This is in agreement with the finding that purely horizontal nystagmus elicited by yaw rotation, strictly in the plane of the horizontal canals, does not induce tilts of the SVV (Pavlou et al. 2003), although the head position required for the present study means that there was a cue conflict between horizontal canals and otoliths.

A study that examined brain responses to both unimodal and combined roll-motion optokinetic and caloric stimulation reported that the combined condition elicited activation reflective of both conditions (Deutschländer et al. 2002), particularly parieto-insular and motion-sensitive visual cortical regions. Of interest, these authors found somewhat less pronounced activation and deactivation during the combined stimulation compared with the single-stimulus conditions (Deutschländer et al. 2002). Although the sign of this visuo-vestibular addition was opposite to that reported here (i.e., our combined caloric-optokinetic stimuli induced larger effects on perceived verticality), when the data were log-transformed we found no difference. This suggests that the internal representation of tilt might follow a logarithm scale, in line with Fechner's law. Both the earlier literature on visual dependence (Witkin 1959; Witkin and Asch 1948) and current studies assessing long-term clinical outcome in patients with acute vestibular lesions indicate that psychological factors (Cousins et al. 2014; Godemann et al. 2004; Lahmann et al. 2015; Staab 2012), notoriously nonlinear, are inextricably linked with visual dependence. Therefore, that we did not observe a direct correlation between baseline VD and torsion during the optokinetic-alone condition is not necessarily surprising. These issues notwithstanding, the findings we present here focus on relating changes in verticality perception to changes in torsional eye movements—effectively normalizing for individual or preexisting differences in VD.

A possible contributory factor that was not explored in this study is the role of vection. Although we could have employed a subjective Likert scale to index vection, a more accurate approach would have required participants to turn a wheel connected to a tachometer as described previously (Okada et al. 1999). However, we decided that this would have interfered with performance of the task and was therefore beyond the scope of this study. Vection might also explain why we observed a larger degree of torsional velocity after caloric compared with optokinetic-alone stimulus but a comparably smaller tilt. We believe this is likely related to differences in perceived direction of self-motion induced by the separate stimuli, with the optokinetic stimulation inducing vection in the same plane the rod is rotated. There is also evidence that the degree of perceived or imagined tilt can also influence estimates of verticality (Barra et al. 2012; Mertz and Lepecq 2001; Zupan and Merfeld 2003); therefore, although the correlations between change in estimated verticality and torsion explain a proportion of the variance, it is likely that the degree of vection or perceived tilt might also have contributed in part to the overall effect.

Caloric stimulation is a potentially useful approach for studying aspects of visuo-vestibular interaction in vestibular neuritis. The caloric response is characterized by prominent horizontal nystagmus with a small torsional component (Fetter et al. 1998; Peterka et al. 2004), similar to vestibular neuritis, although it is worth noting that occasionally in some patients with vestibular neuritis all three canals can be disrupted. When measuring SVV or VD in patients with a resting nystagmus, particularly those with a torsional component (Curthoys et al. 1991), it is important to consider how these eye movements could interact with the task. This finding cannot be explained as simply a perceptual effect since the degree of ocular torsion was also significantly increased, demonstrating that torsional eye movements are an important contributing factor to estimates of verticality. We observed that specific combinations of caloric temperature and visual motion can induce different biases in the perception of verticality; however, these biases only partially canceled out. Despite different temperature calorics inducing biases in opposing directions, the resultant overall increase in tilt is analogous to an increase in VD—as conventionally measured by average tilts induced by both clockwise and anticlockwise disk rotation. Therefore, from an operational perspective overall VD does increase, potentially implying that the effects observed in patient populations are not entirely due to nystagmus (Cousins et al. 2014). In the case of SVV measurements during background roll motion, our data suggest that the presence of a torsional nystagmus can significantly bias the neural mechanisms involved in estimating gravitational vertical; therefore caution should be exercised when drawing conclusions regarding otolith function or VD in the presence of such eye movements. Fundamentally, our results show that, in addition to complex cognitive and perceptual factors, measures of VD are also influenced by basic oculomotor constraints.

## GRANTS

This research was supported by the UK Medical Research Council (MR/J004685/1) and the National Institute for Health Research (NIHR) Imperial Biomedical Research Centre. The views expressed are those of the authors and not necessarily those of the NHS, the NIHR, or the Department of Health.

## DISCLOSURES

No conflicts of interest, financial or otherwise, are declared by the author(s).

## AUTHOR CONTRIBUTIONS

R.E.R., Q.A., and M.P. conception and design of research; R.E.R., M.D.S.M., A.A.S., Q.A., and M.P. performed experiments; R.E.R. and M.P. analyzed data; R.E.R., Q.A., and M.P. interpreted results of experiments; R.E.R. prepared figures; R.E.R. drafted manuscript; R.E.R., M.D.S.M., A.A.S., Q.A., and M.P. edited and revised manuscript; R.E.R., M.D.S.M., A.A.S., Q.A., and M.P. approved final version of manuscript.
